# Peripapillary vessel density correlates with visual field mean sensitivity in highly myopic eyes

**DOI:** 10.1186/s12967-022-03323-9

**Published:** 2022-03-10

**Authors:** Hua Zhong, Qingqing Dong, Qing Cun, Guangyu He, Yijin Tao, Keyao Song, Yunqing Lu, Qin Zhu, Xi Chen, Qin Chen

**Affiliations:** 1 Department of Ophthalmology, The First Affiliated Hospital of Kunming Medical University, Kunming, 650032 China; 2 Department of Ophthalmology, Dazhou Central Hospital, Dazhou, 635099 China; 3 The First People’s Hospital of Yunnan Province, Kunming, 650011 China; 4 Department of Ophthalmology, The Affiliated Hospital of Yunnan University, Kunming, 650051 China; 5 Department of Ophthalmology, The First Affiliated Hospital of Nanjing Medical University, Nanjing, 210000 China

## Abstract

**Purpose:**

To identify the global and regional distribution of peripapillary vessel density (pVD) and try to find out the relationships between pVD and the visual field mean sensitivity (VFMS) in healthy myopic eyes.

**Design:**

Prospective cross-sectional study.

**Methods:**

Two hundred and twenty-two participants (393 eyes) with myopia (myopic refractive error < − 0.5 diopters) from two clinical centers were recruited in this study and were divided into 4 groups according to the spherical equivalent (SE): Group1:− 0.5D ≥ SE > − 6.00D, Group2: − 6.00D ≥ SE > − 8.00D, Group3:− 8.00D ≥ SE > − 10.00D, Group4:SE ≤ -10.00D.The pVD assessed with optical coherence tomography angiography (OCTA) was quantified in 8 sectors. Peripapillary retinal nerve fibre layer (pRNFL) imaging was performed with SD-OCT. Visual field (VF) tests were performed with the 30-2 SITA standard program on the Humphrey 750i Visual Field Analyzer and were grouped into 8 regions that matched the structure.

**Results:**

The pRNFL had no significant difference in all groups (p = 0.422). The average pVD were significantly lower in group 4 (47.61 ± 6.58) than in group 2 and 3 (51.49 ± 3.21, 50.48 ± 3.43 respectively) (p < 0.05). While both pVD in group2 and 3 were statistically lower than group1 (52.77 ± 2.86). The average VFMS was significantly lower in group 4 (901.85 ± 386.54) than other three groups (1169.15 ± 328.94, 1081.77 ± 338.83, 1076.89 ± 358.18, for group1,2,3 respectively). The pVD and VFMS were positively correlated in group3 (r = 0.184) and group4 (r = 0.476) (p < 0.05). Linear regression analysis demonstrated that VFMS were positively associated with pVD especially in temporal and nasal quadrants in myopic eyes.

**Conclusions:**

The pVD shows a significant positive correlation with VFMS in highly myopic eyes with SE ≤ − 8.00D. We suggest that pVD measurement by OCTA could be a sensitive and useful method for monitoring myopic functional change.

## Introduction

Myopia is the most common refractive disorder worldwide and has become one of the leading causes of blindness [1]. By 2050, it is estimated that half of the world's total population (4.758 billion) will suffer from myopia and 938 million people (9.8% of the world's population) will have high myopia [2]. Since high myopia can lead myopic maculopathy and myopia-associated glaucomatous optic neuropathy, it may become one of the main causes for irreversible blindness [2]. Myopic retinopathy, which presents with lacquer crack formation, chorioretinal atrophy, and choroidal neovascularization, is highly correlated with retinal vessel density and morphologic alterations [3]. High myopia has been further reported to be more susceptible to myopia-related structural alterations and to be associated with visual field (VF) impairment [4]. It’s worth noting that there is a relatively high incidence of open angle glaucoma (OAG) in individuals with high myopia, while glaucoma patients represent with optic nerve atrophy and VF damage which may result in the confusion of distinguishing the structural and functional defects caused primarily by myopia or glaucoma [5]. Therefore, studies of the early changes in the structure and function of the optic nerve and retina in myopic patients, as well as the relationship between them, may have great significance in evaluating the development and prognosis of myopia-related optic nerve and retinal abnormalities and in advancing new strategies for the prevention and treatment of high myopia. Furthermore, a correct understanding of the relationship between structural and functional damage helps to accurately distinguish glaucomatous optic neuropathy from high myopia [5].

Myopia can significantly affect the distribution and thickness of retinal nerve fiber layer (RNFL). Structural assessment of high myopia is complicated due to paraoptic disc atrophy, optic disc tilt, biomechanical stretch and deformation of peripapillary retinal nerve fiber layer (pRNFL), and axial stretch of eyeball [6]. Many studies have used optical coherence tomography(OCT) such as cirrus HD-OCT or Stratus OCT technology to measure pRNFL of myopic patients, but the results of these studies are influenced by various ocular structure changes in myopic eyes [7–9]. The prevalence of disc edge detection errors in myopic eyes ranged from 0.5% to 17.6%, and the incidence of cup detection errors was 1.1% to 7.5% [10, 11]. Meanwhile the segmentation algorithms of OCT are tailored for normal retinal anatomy, variations from this normal in form of high refractive errors raises pertinent concerns regarding accuracy and reliability of OCT in these cases. These situations limited the use of OCT in high myopic eyes by affecting scan acquisition and data interpretation, thus leading to mistaken diagnosis.

Radial peripapillary capillaries (RPCs) comprise a unique network of capillary beds within the RNFL, while the average RNFL is positively correlated with the vessle density (VD) of RPCs [12, 13]. The recent use of optical coherence tomography angiography (OCTA) has enabled non-invasive assessments of the microvasculature in the optic nerve head (ONH), RPCs and macula. OCTA has the advantage of being unaffected by retinal nerve fiber hyporeflexia or structural deformation of the optic nerve (e.g., optic disc tilt or paraoptic disc atrophy) [14, 15]. There have been a number of studies of the repeatability and reproducibility of OCTA not only in normal eyes but also in myopic eyes, and the results showed high repeatability [16]. In previous OCTA studies in eyes with high myopia, evidence of abnormal peripapillary and macular microvasculature has been observed [17–20]. However, the association between VD and VF sensitivity and whether if it consistent with the alteration of RNFL thickness in high myopic patients has not been reported before.

The purpose of this study was to identify the global and regional distribution of peripapillary vessel density (pVD) using OCTA and try to find out the relationships between pVD and the visual field mean sensitivity (VFMS) assessed by standard automated perimetry (SAP), and then compare them with the associations between peripapillary RNFL (pRNFL) thickness and VFMS in healthy myopic eyes. Through this study, we tried to explore an effective and noninvasive method for monitoring functional change in highly myopic eyes.

## Methods

### Subjects

This multicenter prospective cross-sectional study enrolled myopic subjects from the First Affiliated Hospital of Kunming Medical University and the First Affiliated Hospital of Nanjing Medical University, China, in a consecutive manner between August 2019 and January 2021. Two hundred and twenty-two participants (393 eyes), 18 to 60 years old, who were diagnosed as myopia (the spherical equivalent (SE) was − 0.5 diopters (D) or less) were recruited in this study. Ethical approval for the study was obtained from the Institutional Review Board of Kunming Medical University and the First Affiliated Hospital of Nanjing Medical University. This project was conducted in line with the principles of the Helsinki Declaration. Written informed consent was obtained from each participant.

The following conditions were excluded: (1) Long-term ocular or systemic use of corticosteroids; (2) patients with glaucoma or suspected glaucoma (including normal tension glaucoma, NTG) with focal or diffuse optic disc neuroretinal rim loss; (3) intraocular pressure (IOP) > 21 mmHg; (4) abnormalities or diseases may cause visual field defects such as disc drusen, staphyloma, optic neuritis, ischemic optic neuropathy, optic papilledema, retinal vessel occlusion, retinal detachment, macular disease, etc. (5) history of previous ocular surgery, such as vitreoretinal surgery, glaucoma surgery, cataract surgery or LASIK surgery, that may affect the refractive status.

Both eyes of each participant were included, unless one eye did not meet the above inclusion–exclusion criteria. In our study, the enrolled subjects were divided into 4 groups according to the SE: Group 1: − 0.5D ≥ SE > − 6.00D, Group 2: − 6.00D ≥ SE > − 8.00D, Group 3: − 8.00D ≥ SE > − 10.00D, Group 4: SE ≤ − 10.00D. All participants underwent a complete ophthalmic evaluation, including manifest refractive error (spherical equivalent), best-corrected visual acuity (BCVA), IOP measurements with Goldmann applanation tonometer, and detailed ophthalmoscopic examinations. The BCVA measured using a decimal VA chart was converted to the logarithm of the minimum angle of resolution (logMAR) VA. Biometric measurements anterior chamber depth (ACD), and axial length (AL) of each eye were taken using the IOL master 500 (Carl Zeiss Jena, Germany). The central corneal thickness (CCT) was measured by ultrasonic pachymetry.

Automated visual field tests were performed with the 30-2 SITA standard program on the Humphrey 750i Visual Field Analyzer (Carl Zeiss Meditec, Dublin, CA, USA). For each visual field, we evaluated the mean deviation (MD) and the pattern standard deviation (PSD). Data of 54 locations in the visual field corresponding to the test sites of the Humphrey 24-2 SITA standard program was also analyzed. The first VF test was excluded to minimize the learning effect. Reliable visual field tests were defined as follows: fixation loss rate < 20%, false positive rate < 15%, false negative rate < 15%.

### RNFL imaging

RNFL imaging was performed with the SD-OCT device (RTVue, XR, Avanti, Optuovue, Inc., Fremont, CA, USA) using a scanning laser diode to emit a scan beam with a wavelength of 840 nm to provide images of ocular microstructures [21]. Using the 4.5-mm diameter RTVue protocols that calculates the peripapillary retinal nerve fibre layer (pRNFL) thickness in a 4.5 mm diameter circle centered on the ONH. Criteria for determining scan quality were signal strength of 30 or more, a clear fundus image allowing optic disc and the scan circle visibility before and during image acquisition, and absence of scan failures and *en face* OCT (“*en face*” is an emerging imaging technique derived from spectral domain OCT which produces frontal sections of retinal layers, also called "C-scan OCT”) image distortions because of blinking or eye movements.

### OCTA imaging

**Fig. 1:**
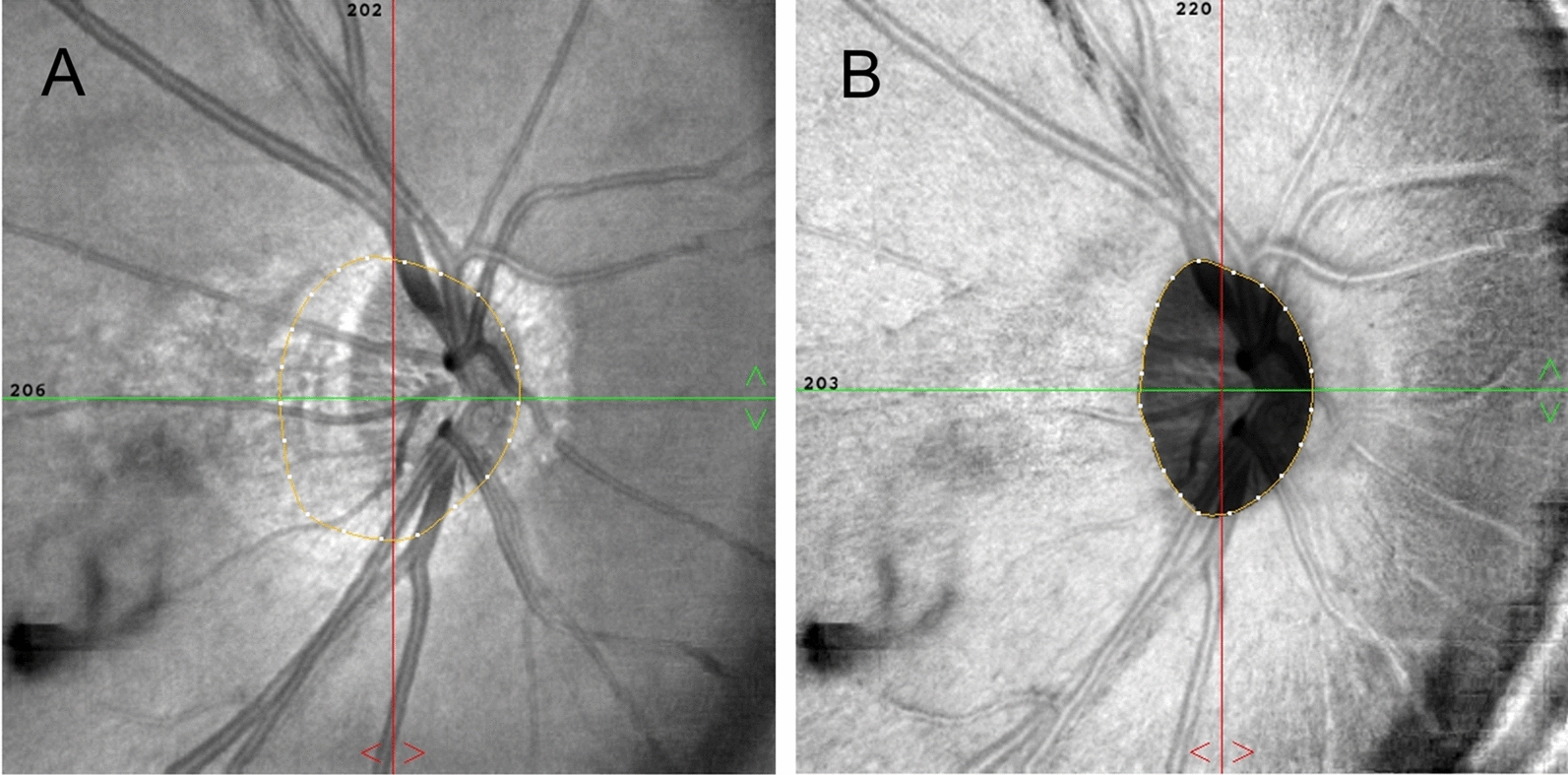
Optic disc boundary in optical coherence tomography (OCT) and optical coherence tomography angiography (OCTA). **A** The boundary of the optic disc was labelled automatically. **B** The boundary of the optic disc was then manually delineated, and the center of the optic disc was determined by automatically finding the best ellipse fit of the optic disc boundary

The OCTA examination was performed with the participants under pharmacological mydriasis. OCT-A images were obtained with a commercial spectral-domain OCT system (RTVue, XR, Avanti, Optuovue, Inc., Fremont, CA, USA), with a scan rate of 70,000 A-scans per second, scan beam wavelength centered at 840 nm, and a bandwidth of 45 nm. The cube scans consisted of 73 B-scans comprised of 768 A-scans each captured in a 4.5 × 4.5 mm square centered on the ONH. Capillary densities from the internal limiting membrane (ILM) to the RNFL posterior boundary are imaged by the standard AngioVue software, and this measurement has been termed the RPC density image. Capillary density was measured with a built-in feature called large-vessel masking, which removes vessels with a diameter of ≥ 3 pixels, approximately ≥ 33 µm for the 4.5-mm AngioVue optic disc (AngioDisc) scans (Optuovue 2018.1.1.63 software version) [22]. The vessel density (VD), defined as the percentage area occupied by microvasculature, was evaluated in the 4.5 × 4.5-mm scan area excluding the central 2 mm diameter circle, which was manually centered on the optic disc based on the *en face* reflectance image [23]. The boundary of the optic disc was then manually delineated, and the center of the optic disc was determined by automatically finding the best ellipse fit of the optic disc boundary (Fig. 1). The split-spectrum amplitude-decorrelation angiography (SSADA) algorithm was implemented to enhance visualization of the OCTA images (304 × 304 pixels). Poor quality scans were excluded from the analysis if one of the following criteria were met: (1) poor clarity images; (2) local weak signal caused by artifacts such as floaters; (3) residual motion artifacts visible as irregular vessel patterns on the en face angiogram and (4) scans with segmentation failure.

**Fig. 2:**
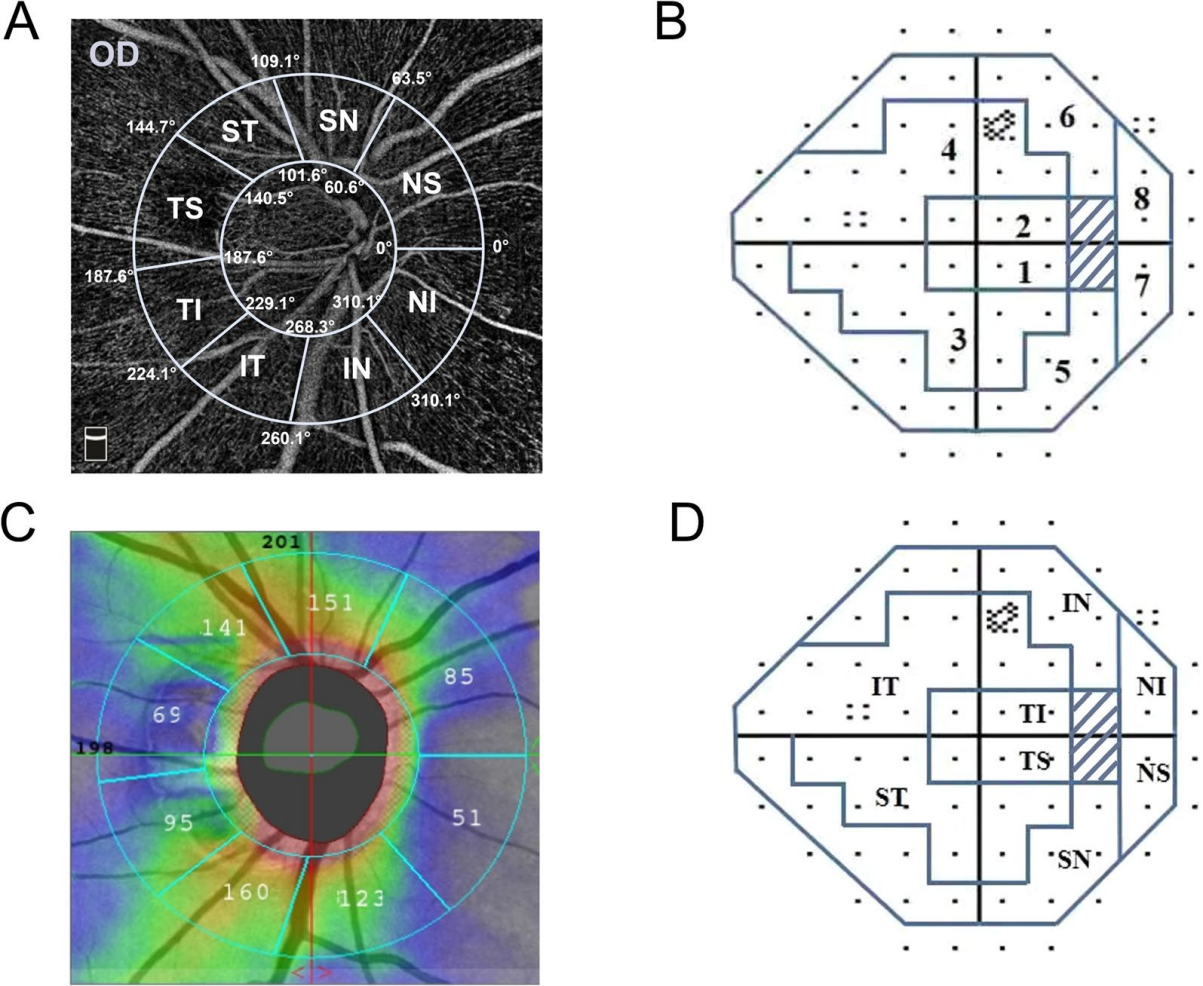
Eight peripapillary structure and function regions. **A** The peripapillary region of optical coherence tomography angiography (OCTA) is divided into eight sectors in accordance with the regionalization previously described by Garway-Heath. **B** Fifty-four locations in the 30–2 SITA-standard visual field test corresponding to the test sites of the 24-2 SITA standard program were included and were divided into 8 sectors according to Garway-Heath scheme. **C** 4.5 × 4.5-mm *en face* optical coherence tomography (OCT) of the peripapillary retinal nerve fibre layer (pRNFL) and was divided into 8 zones automatically. **D** The structural correspondence zone on the 30-2 visual field test. TS: temporal superior; TI: temporal inferior; ST: superior temporal; IT: inferior temporal; SN: superior nasal; IN: inferior nasal; NS: nasal superior; NI: nasal inferior

The peripapillary region is defined as a 1-mm wide elliptical annulus extending from the ONH boundary [24]. This peripapillary region is divided into eight sectors in accordance with the regionalization previously described by Garway-Heath et al. [25]: ① temporal superior (TS)187.6° to 144.7°, ② temporal inferior (TI) 224.1° to 187.6°, ③ superior temporal (ST) 144.7° to 109.1°, ④ inferior temporal (IT) 260.1° to 224.1°, ⑤ superior nasal (SN) 109.1° to 63.5°, ⑥ inferior nasal (IN) 310.1° to 260.1°, ⑦ nasal superior (NS) 63.5° to 0°, and ⑧ nasal inferior (NI) 0° to 310.1°. VF testing points were grouped into eight regions that matched the structure- function map (Fig. 2) [16].

### Data processing of pRNFL and pVD

It has been found that actual scanning radius in eyes with greater axial length (myopic eyes) could be longer than 1.73 mm due to magnification effect, so using same-sized scan circle for measuring pRNFL in patients with different degrees of myopia might be misleading because the RNFL thickness decreases with increasing distances from the optic disk [26]. Therefore, the pRNFL results of the patients with myopia need to be adjusted according to the axial length (AL). In this study, Bennett's amplification formula was used for the correction of AL-related ocular magnification effects. The relationship between the OCT measurements and the actual scan diameter was expressed by the formula t = p × q × s, where t was the actual fundus dimension, p was the magnification factor determined by the camera of the OCT imaging system, q represented the magnification factor in relation to the eye, and s was the original measurement obtained from the OCT. The correction factor q was determined with the equation q = 0.01306 × (AL − 1.82) in this study [27]. The data of pRNFL in this study were corrected with this formula. When it comes to pVD, which represented the percentage area occupied by the microvasculature, was less influenced by AL-related magnification effects than that of pRNFL. Therefore, we didn’t use any amplification formula to correct the data of pVD.

### Data processing of VFMS

The VFMS at various VF sectors was defined as the average value of the differential light sensitivity (DLS) obtained at VF test locations corresponding to pVD sectors. The DLS of visual field cursor is measured and expressed in logarithmic decibels (dB). If the correlation between dB value and pVD or pRNFL was analyzed directly, the logarithmic curve relationship was obtained [28]. According to the formula dB = 10 × log_10_ (1/L), divide the dB value by 10, and then calculate the inverse number to get 1/L (1/L represents the maximum brightness of the instrument/the brightness of the visual object actually detected, L: luminance measured in Lamberts) [6, 29]. After calculating the 1/L of each point, the average value of each area was calculated according to the eight sectors we have described above. Then we got the data of VFMS which was expressed in unlogged 1/L scales. The relationships between the structure and function of myopia were analyzed by comparing the pVD or pRNFL to the corresponding VFMS.

### Statistical analysis

All statistical analyses were performed using the SPSS software version 23.0 (SPSS Inc,Chicago, Illinois, USA). (1) By K-S single sample test, the population was normal distribution, the four groups of data were respectively used descriptive statistics, the variables were expressed as mean ± standard deviation (SD). Categorical variables were compared using Chi-square test between the groups. One-way ANOVA with Dunnett's multiple comparisons test was used to determine significance between groups for normal distribution parameters. For non-normal distribution parameters, the Kruskal–Wallis H test (k independent samples) was used to compare groups of variants. When p < 0.05, the difference was statistically significant. (2) The correlation between pRNFL, pVD and VFMS was analyzed using the Pearson correlation co-efficient (r). (3) The univariate and multivariate linear regression analyses were performed to examine the associations between pRNFL, pVD and VFMS in each sector.

## Results

### Demographic and clinical characteristics of patients in four groups

A total of 52 patients (89 eyes) with mild to moderate myopia (from − 0.5D to − 5.75D) and 170 patients (304 eyes) with high myopia (≤ − 6.00D) were included in this study. The average age was 32.05 ± 10.11 years (range: 18–60 years). There were 73 males and 149 females. Table 1 summarizes and compares the demographic and clinical characteristics of the subject eyes of all the four groups. Gender, age, IOP and CCT were similar between the four groups (p > 0.05). There were statistically significant differences in BCVA, AL, MD and PSD of visual field among the four groups (p < 0.05). The MD of visual field was decreased gradually from group 1 to group 4. The average PSD of visual field was significantly higher in group 4 than in other three groups.

**Table 1:** Demographic and clinical characteristics of subjects in 4 groups

	Group1 (n = 89)	Group2 (n = 102)	Group3 (n = 122)	Group4 (n = 80)	P value	Post-hoc test (group)
Sex (male:female)^a^	30:59	34:68	33:89	29:51	0.525	**–**
Age (years)	30.05 ± 10.32	31.9 ± 9.27	32.40 ± 9.54	33.89 ± 11.43	0.960	**–**
BCVA (log MAR)	− 0.015 ± 0.09	0.007 ± 0.05	0.009 ± 0.05	0.078 ± 0.15	< 0.001^*^	**4 < 3,2 < 1**
Intraocular pressure (IOP, mmHg)	15.40 ± 2.56	16.13 ± 2.00	16.51 ± 2.23	16.18 ± 2.74	0.110	**–**
Axial length (AL, mm)	24.96 ± 0.96	26.21 ± 0.77	26.84 ± 0.95	27.97 ± 1.36	< 0.001^*^	**4 > 3 > 2 > 1**
Central corneal thickness (CCT, μm)	522.67 ± 33.58	517.0 ± 26.49	520.31 ± 32.55	521.23 ± 30.61	0.611	**–**
Visual field, MD (dB)	− 1.98 ± 1.23	− 2.46 ± 1.63	− 2.60 ± 1.53	− 3.53 ± 2.30	< 0.001^*^	**4 < 3,2 < 1**
Visual field, PSD(dB)	2.23 ± 1.00	2.32 ± 0.96	2.57 ± 1.24	3.28 ± 1.77	< 0.001^*^	**4 > 3,2,1**

^a^Categorical variables were compared using Chi-square test

One-way ANOVA with Dunnett's multiple comparisons test was used to determine significance between groups

^*^p < 0.05, the difference is statistically significant. Significant values are in bold type

### Results of pRNFL, pVD and VFMS assessments

**Fig. 3:**
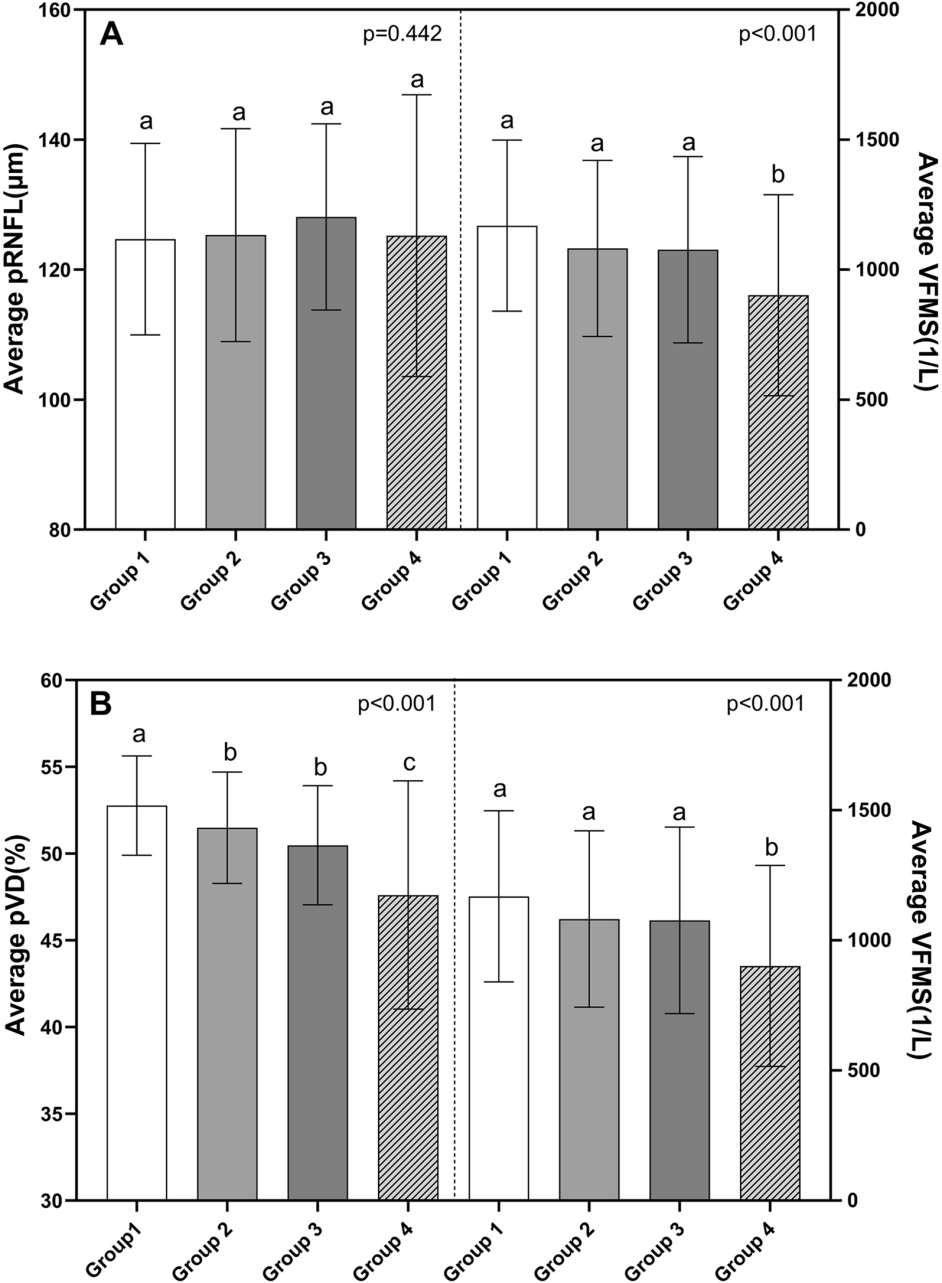
The average level of pRNFL, pVD and VFMS in four groups and the comparisons between groups. The average level of the peripapillary retinal nerve fibre layer (pRNFL) thickness assessed by optical coherence tomography (OCT) showed no statistically difference between four groups (the left part of (**A**). The average level of visual filed mean sensitivity (VFMS) was significantly lower in group 4 than in group 1,2 and 3(the right part of **A**). Peripapillary vessel density (pVD) assessed by optical coherence tomography angiography (OCT-A) showed a deceasing trend from group 1 to 4(the left part of **B**). a, b, c: statistical differences between groups were marked by letters, with groups sharing at least one similar letter presenting no statistically significant difference (p ≥ 0.05) and groups with all different letters presenting a significant difference (p < 0.05)

**Fig. 4:**
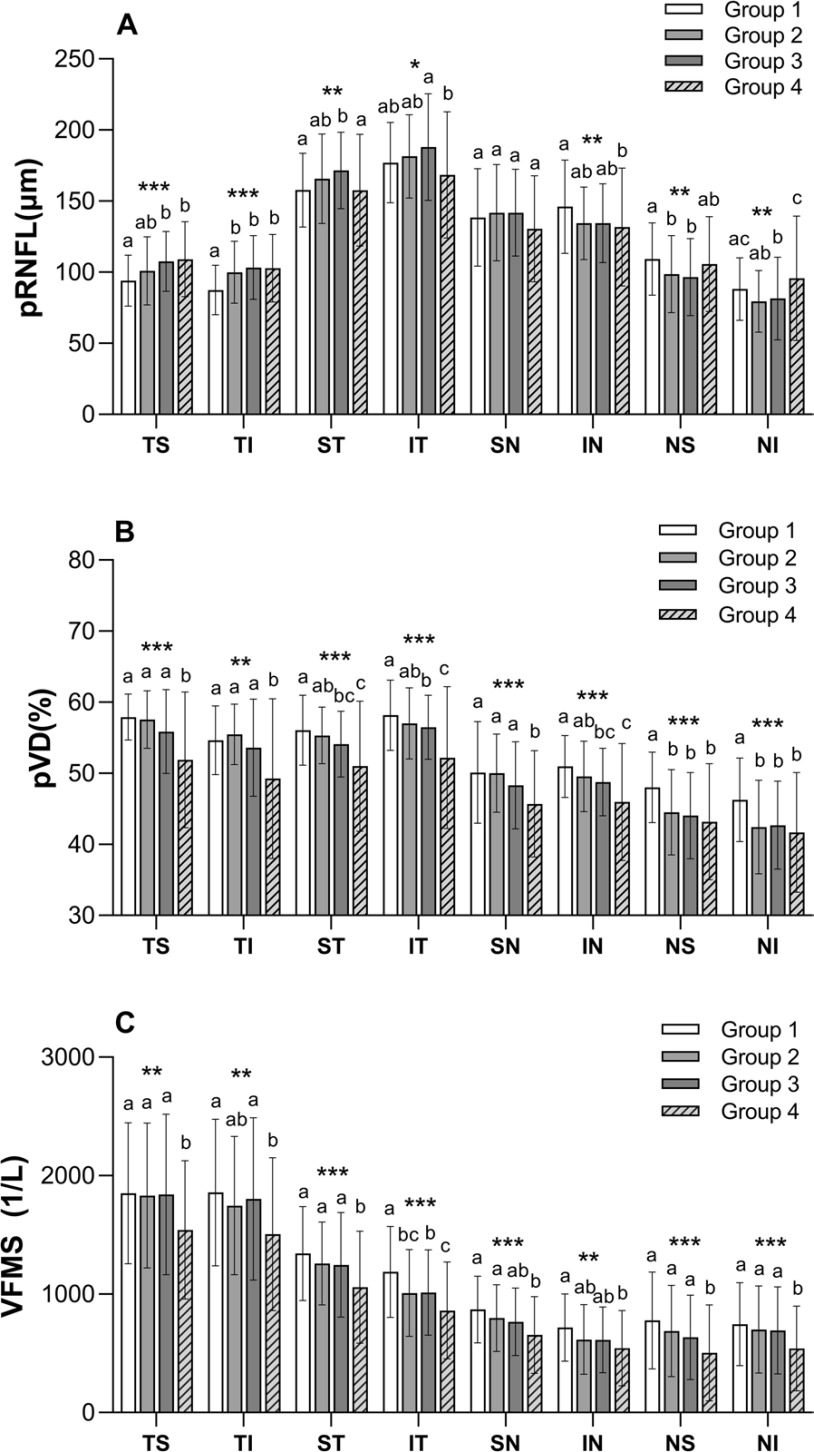
The level of pRNFL, pVD and VFMS in 8 peripapillary regions among four groups. The regional level of the peripapillary retinal nerve fibre layer (pRNFL), peripapillary vessel density (pVD) and visual filed mean sensitivity (VFMS) were showed separately in **A–C**. **A** The TS and TI regional pRNFL in Group 3 and 4 were statistically significant thicker than that in Group1. However, in IT and IN region, the pRNFL in Group 4 was thinner than that in Group 3 and Group1, separately. **B** The regional pVD decreased along with the refractive error diopters increased. This decline was particularly pronounced in Group 4. **C** The regional VFMS decreased significantly in Group 4 in all the 8 peripapillary areas. * P < 0.05, ** P < 0.01, ***P < 0.001(comparison in each sector between four groups). a, b, c: statistical differences between groups were marked by letters, with groups sharing at least one similar letter presenting no statistically significant difference (p ≥ 0.05) and groups with all different letters presenting a significant difference (p < 0.05). TS: temporal superior; TI: temporal inferior; ST: superior temporal; IT: inferior temporal; SN: superior nasal; IN: inferior nasal; NS: nasal superior; NI: nasal inferior

The pRNFL calculated are summarized in Table 2. Although the average pRNFL had no significant difference in all groups (p = 0.422), the TS and TI regional pRNFL in highly myopic eyes (group3,4) were thicker than low to moderate myopic eyes (group 1). While in the nasal sectors, the situation was quite different. In IN region, the pRNFL in group 4 (118.8 μm) was thinner than that in group 1 (139.9 μm). In NS sector, the pRNFL in group 2 and 3 (96.4 μm and 93.6 μm) was also thinner than that in group 1 (110.1 μm). Table 3 summarizes and compares the average and regional pVD percentage. Both global and regional pVD were significantly different among the four groups (p < 0.05). The average pVD was decreased as the negative refractive error increased. The comparisons of average and sectoral VFMS are displayed in Table 4. The average VFMS demonstrated a progressive decrease from group 1,2,3 (1169.15 ± 328.94, 1081.77 ± 338.83, 1076.89 ± 358.18, respectively) to group 4(901.85 ± 386.54). At the same time, there were significant differences of VFMS in every single zone among the four groups (p < 0.05). The results of average level of pRNFL, pVD and VFMS in four groups and the comparisons between groups are showed in Fig. 3. And the sectorial level of pRNFL, pVD and VFMS among four groups are showed in Fig. 4.

**Table 2:** Average and regional peripapillary retinal nerve fibre layer (pRNFL) thickness in each group and the comparisons between four groups

	Group1 (n = 89)	Group2 (n = 102)	Group3 (n = 122)	Group4 (n = 80)	P value	Pair comparison (group)
Average pRNFL (μm)	124.69 ± 14.72	125.32 ± 16.39	128.10 ± 14.33	125.24 ± 21.67	0.422	–
TS pRNFL (μm)	90.9 (81.9–104.4)	101.1 (88.3–108.6)	106.7 (91.8–118.8)	106.1 (88.1–124.6)	< 0.001^*^	**4,3 > 1**
TI pRNFL (μm)	86.1 (76.5–98.7)	96.2 (85.1–113.0)	99.7 (89.2–119.5)	100.6 (84.1–120.7)	< 0.001^*^	**4,3,2 > 1**
ST pRNFL (μm)	157.9 (143.5–172.5)	161.2 (145.0–178.0)	168.1 (154.0–186.2)	158.3 (132.2–182.7)	0.003^*^	**3 > 4,1**
IT pRNFL (μm)	181.9 (158.9–196.8)	177.9 (161.8–198.5)	184.2 (164.9–213.4)	170.8 (142.6–197.2)	0.019^*^	**4 < 3**
SN pRNFL (μm)	140.6 (124.2–162.8)	138 (116.4–165.6)	139.6 (116.2–165.5)	124.3 (106.7–154.6)	0.079	–
IN pRNFL (μm)	139.9 (126.7–160.4)	132.4 (115.9–149.3)	131.8 (116.8–152.1)	118.8 (100.4–160.1)	0.008*	**4 < 1**
NS pRNFL (μm)	110.1 (92.0–121.6)	96.4 (79.3–116.8)	93.6 (78.3–109.2)	103.2 (86.7–120.6)	0.001^*^	**2,3 < 1**
NI pRNFL (μm)	86.6 (72.9–100.9)	77.7 (61.7–94.9)	73.9 (61.5–93.3)	87.1 (69.9–112.0)	0.001^*^	**4 > 2,3;3 < 1**

One-way ANOVA with Dunnett's multiple comparisons test was used to determine significance between groups for normal distribution parameters, and the results were documented as mean ± SD. For non-normal distribution parameters, the Kruskal–Wallis H test (k independent samples) was used to compare groups of variants, and the results were documented as median (lower and upper quartile)

TS, temporal superior; TI, temporal inferior; ST, superior temporal; IT, inferior temporal; SN, superior nasal; IN, inferior nasal; NS, nasal superior; NI, nasal inferior

^*^p < 0.05, the difference is statistically significant and significant values are in bold type

**Table 3:** Average and regional peripapillary vessel density (pVD) percentage in each group and the comparisons between four groups

	Group1 (n = 89)	Group2 (n = 102)	Group3 (n = 122)	Group4 (n = 80)	P value	Pair comparison (group)
Average pVD (%)	52.77 ± 2.86	51.49 ± 3.21	50.48 ± 3.43	47.61 ± 6.58	< 0.001*	**4 < 3,2 < 1**
TS pVD (%)	58 (56–60)	58 (55–60)	57 (53–60)	54 (47.3–58)	< 0.001*	**4 < 3,2,1**
TI pVD (%)	55 (52–57)	56 (52–58)	55 (51–58)	52 (45.3–58)	0.001^*^	**4 < 3,2,1**
ST pVD (%)	57 (53.5–59)	55 (53–58)	54 (51–57)	53 (47–57.8)	< 0.001*	**4,3 < 1; 4 < 2**
IT pVD (%)	59 (56–62)	57.5 (54–60)	57 (54–59)	54 (49–58)	< 0.001*	**4 < 3,2,1; 3 < 1**
SN pVD (%)	51 (47–55)	50 (46–54)	49 (45.8–52)	46 (41–50)	< 0.001*	**4 < 3,2,1**
IN pVD (%)	51 (47–54)	49 (46–53)	50 (45.8–52)	47 (42.3–50.8)	< 0.001*	**4 < 2,1; 3 < 1**
NS pVD (%)	49 (45.5–51)	45 (40.8–49)	45 (40–48)	44.5 (39–49)	< 0.001*	**4,3,2 < 1**
NI pVD (%)	48 (43–50)	42 (38–47)	43 (39–46.3)	44 (36.3–47)	< 0.001*	**4,3,2 < 1**

One-way ANOVA with Dunnett's multiple comparisons test was used to determine significance between groups for normal distribution parameters, and the results were documented as mean ± SD. For non-normal distribution parameters, the Kruskal–Wallis H test (k independent samples) was used to compare groups of variants, and the results were documented as median (lower and upper quartile)

TS, temporal superior; TI, temporal inferior; ST, superior temporal; IT, inferior temporal; SN, superior nasal; IN, inferior nasal; NS, nasal superior; NI, nasal inferior

^*^p < 0.05, the difference is statistically significant and significant values are in bold type

**Table 4:** Average and regional visual field mean sensitivity (VFMS) in each group and the comparisons between four groups

	Group1 (n = 89)	Group2 (n = 102)	Group3 (n = 122)	Group4 (n = 80)	P value	Pair comparison (group)
Average	1169.2 ± 328.9	1081.8 ± 338.8	1076.9 ± 358.2	901.9 ± 386.5	< 0.001^*^	**4 < 3,2,1**
Zone 1	1721.7 (1418.1–2167.5)	1776.2 (1411.0–2167.5)	1749.8 (1356.0–2176.3)	1490.3 (1086.3–2030.7)	0.007^*^	**4 < 3,2,1**
Zone 2	1698.9 (1458.2–2167.5)	1687.8 (1384.3–2167.5)	1721.7 (1281.3–2187.5)	1438.1 (1121.2–1922.0)	0.009^*^	**4 < 3, 1**
Zone 3	1342.9 ± 395.4	1257.9 ± 349.6	1246.4 ± 441.3	1058.7 ± 472.9	< 0.001^*^	**4 < 3,2,1**
Zone 4	1129.5 (901.9–1432.2)	937.6 (725.4–1276.3)	1014.0 (779.2–1293.1)	792.32 (561.8–1183.3)	< 0.001*	**4 < 3 < 1; 2 < 1**
Zone 5	822.3 (665.2–1058.3)	764.6 (595.8–940.7)	740.3 (578.8–967.2)	617.4 (377.3–862.1)	< 0.001*	**4 < 2,1**
Zone 6	685.5 (513.8–884.8)	590.5 (374.2–778.6)	588.7 (398.5–831.7)	450.7 (306.0–766.1)	0.001^*^	**4 < 1**
Zone 7	712.6 (473.6–1013.3)	647.8 (392.6–897.2)	566.1 (392.6–897.2)	398.0 (204.1–712.6)	< 0.001*	**4 < 3,2,1**
Zone 8	647.8 (501.2–972.5)	643.6 (449.6–835.9)	639.4 (447.5–897.2)	473.6 (232.3–712.6)	0.001*	**4 < 3,2,1**

One-way ANOVA with Dunnett's multiple comparisons test was used to determine significance between groups for normal distribution parameters, and the results were documented as mean ± SD. For non-normal distribution parameters, the Kruskal–Wallis H test (k independent samples) was used to compare groups of variants, and the results were documented as median (lower and upper quartile)

^*^p < 0.05, the difference is statistically significant and significant values are in bold type

### Correlations between structure (pRNFL, pVD) and function (VFMS)

**Fig. 5:**
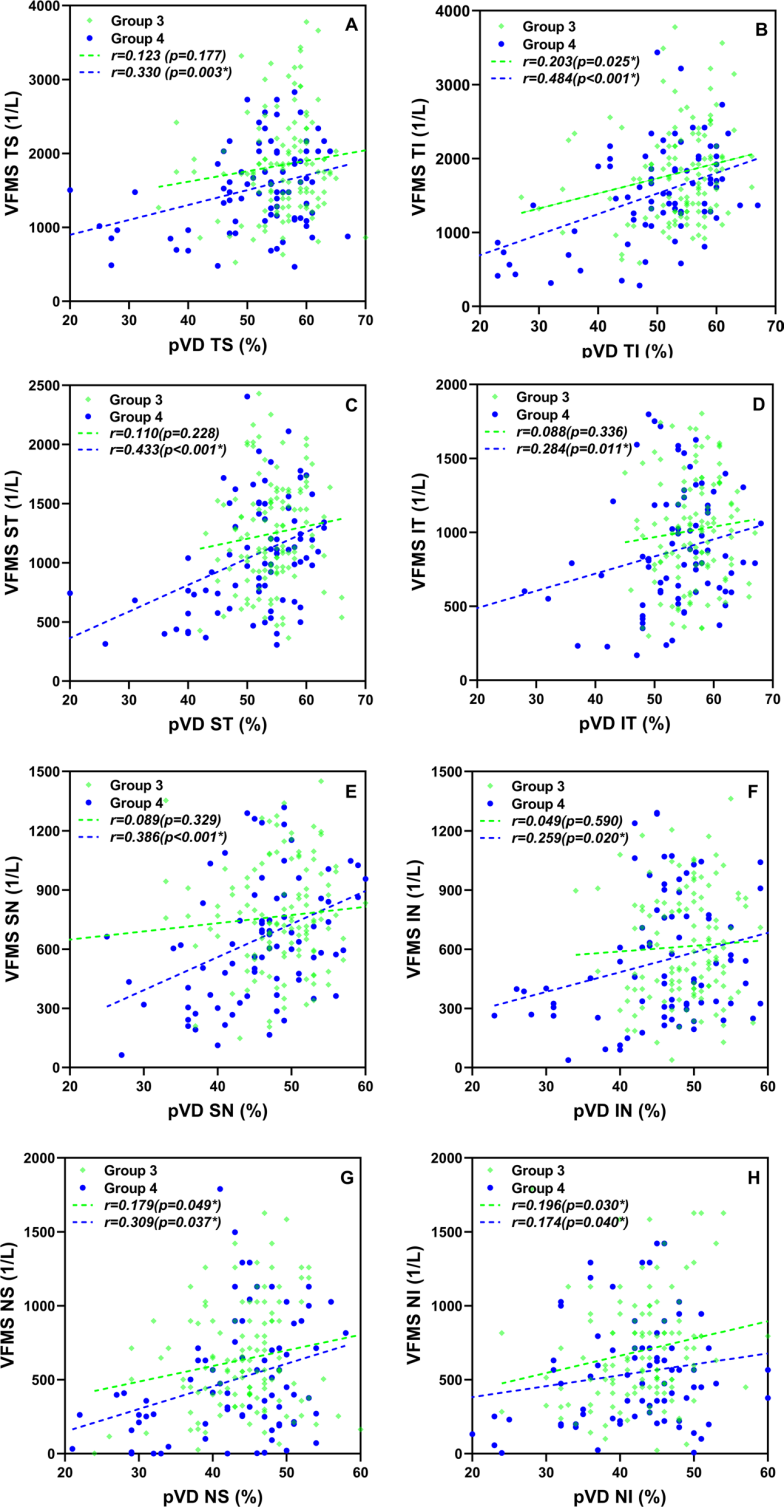
Regional relationships between pVD and corresponding VFMS in Group 3 and Group 4. Scatter plots of the regional peripapillary vessel density (pVD) and corresponding visual field mean sensitivity (VFMS) in Group 3 and Group 4. The pVD-VFMS associations were significant in all the sectors in Group 4 (p < 0.05). While in Group 3, there were significant correlations only in the TI, NS and NI sectors. **A** The pVD-VFMS correlation in TS region was only significant in Group 4 (p=0.003). **B** The pVD-VFMS correlations in TI region were significant in both Group 3(p=0.025) and Group 4 (p<0.001). **C** The pVD-VFMS association in ST region was statistically significant in Group 4(p<0.001), while not significant in Group 3. **D** The pVD-VFMS association in IT region was statistically significant only in Group 4(p=0.011). **E** The pVD-VFMS correlation in SN region was only significant in Group 4 (p<0.001). **F** The pVD-VFMS association in IN region was statistically significant only in Group 4 (p=0.020). **G** Correlations between pVD and VFMS in NS region were statistically significant in both Group 3 (p=0.049) and Group 4 (p=0.037). **H** The pVD-VFMS correlations in NI region were significant in both Group 3(p=0.030) and Group 4(p=0.040).TS, temporal superior; TI, temporal inferior; ST, superior temporal; IT, inferior temporal; SN, superior nasal; IN, inferior nasal; NS, nasal superior; NI, nasal inferior. p < 0.05, the difference was statistically significant

The results of structure and function correlation among the four groups are shown in Table 5. There was no significant correlation between the average pRNFL and VFMS in all groups (p > 0.05), while the average pVD and VFMS in group 3 (r = 0.184) and group 4 (r = 0.476) were positively correlated (p < 0.05). In regional analysis, there were no statistically significant correlations found between pRNFL and VFMS in all the eight sectors in eyes with SE >− 10.00D regardless of location other than the nasal-superior sector in group 2 (r = 0.206, p = 0.014). In the temporal-superior sector, pVD and VFMS was positively correlated only in eyes SE ≤− 10.00D (r = 0.330, p < 0.001). The regional associations between pVD and VFMS were significant at the temporal-inferior, nasal-superior and nasal-inferior sectors (corresponding to the functional zone 2,7 and 8) in eyes of group 1, 3 and 4. Whereas, there were no relationships were observed between pRNFL and VFMS at the three regions except the nasal-inferior sector where a negative correlation was found in group 4. When it comes to the superior-temporal, inferior-temporal, superior-nasal and inferior-nasal sectors, which correspondence to functional zones 3,4 5 and 6, no significant relationships were found between structure and function in eyes with SE > − 10.00D. But in eyes with SE ≤ − 10.00D, significant positive correlations were found between pVD and pRNFL at the four sectors mentioned above. Actually, the pVD-VFMS association was significant in all the sectors in group 4 (p < 0.05). The regional association between the pVD and VFMS was significantly stronger than that of pRNFL and VFMS especially in group 4. Figure 5 shows regional relationships between pVD and corresponding VFMS in group 3 and group 4.

**Table 5:** The correlation analyses between peripapillary vessel density (pVD) and visual filed mean sensitivity (VFMS), peripapillary retinal nerve fibre layer (pRNFL) and VFMS within groups

	Group1 (n = 89)		Group2 (n = 102)		Group3 (n = 122)		Group4 (n = 80)	
	pRNFL-VFMS	pVD-VFMS	pRNFL-VFMS	pVD-VFMS	pRNFL-VFMS	pVD-VFMS	pRNFL-VFMS	pVD-VFMS
	r(p)	r(p)	r(p)	r(p)	r(p)	r(p)	r(p)	r(p)
Average	− 0.090 (0.402)	− 0.096 (0.371)	0.002 (0.985)	− 0.029 (0.771)	0.020 (0.830)	0.184 (0.042^*^)	0.067 (0.555)	0.476 (< 0.001^*^)
TS	− 0.520 (0.627)	− 0.058 (0.590)	− 0.167 (0.094)	− 0.099 (0.320)	0.036 (0.698)	0.123 (0.177)	− 0.170 (0.132)	0.330 (0.003^*^)
TI	− 0.003 (0.978)	0.270 (0.010^*^)	− 0.099 (0.324)	− 0.095 (0.342)	0.096 (0.291)	0.203 (0.025^*^)	− 0.121 (0.287)	0.484 (< 0.001^*^)
ST	0.109 (0.311)	− 0.006 (0.956)	− 0.033 (0739)	0.143 (0.152)	0.035 (0.701)	0.110 (0.228)	0.228 (0.042^*^)	0.433 (< 0.001^*^)
IT	0.142 (0.185)	− 0.021 (0.844)	0.004 (0.968)	− 0.078 (0.438)	0.115 (0.209)	0.088 (0.336)	0.369 (0.001^*^)	0.284 (0.011^*^)
SN	− 0.149 (0.164)	− 0.115 (0.284)	0.037 (0.711)	0.01 0(0.919)	− 0.018 (0.846)	0.089 (0.329)	0.169 (0.134)	0.386 (< 0.001^*^)
IN	0.164 (0.124)	0.111 (0.299)	0.178 (0.073)	0.022 (0.829)	0.048 (0.598)	0.049 (0.590)	0.024 (0.835)	0.259 (0.020^*^)
NS	0.038 (0.721)	0.261 (0.014^*^)	0.206 (0.038^*^)	0.192 (0.053)	0.025 (0.786)	0.179 (0.049^*^)	0.043 (0.704)	0.309 (0.037^*^)
NI	0.094 (0.381)	0.249 (0.019^*^)	0.114 (0.225)	0.078 (0.438)	0.031 (0.736)	0.196 (0.030^*^)	0.26 (0.017^*^)	0.174 (0.040^*^)

r: Pearson correlation coefficient

TS, temporal superior; TI, temporal inferior; ST, superior temporal; IT, inferior temporal; SN, superior nasal; IN, inferior nasal; NS, nasal superior; NI, nasal inferior

^*^p < 0.05, the correlation is significant and significant values are in bold type

### Linear regression analysis

**Fig. 6:**
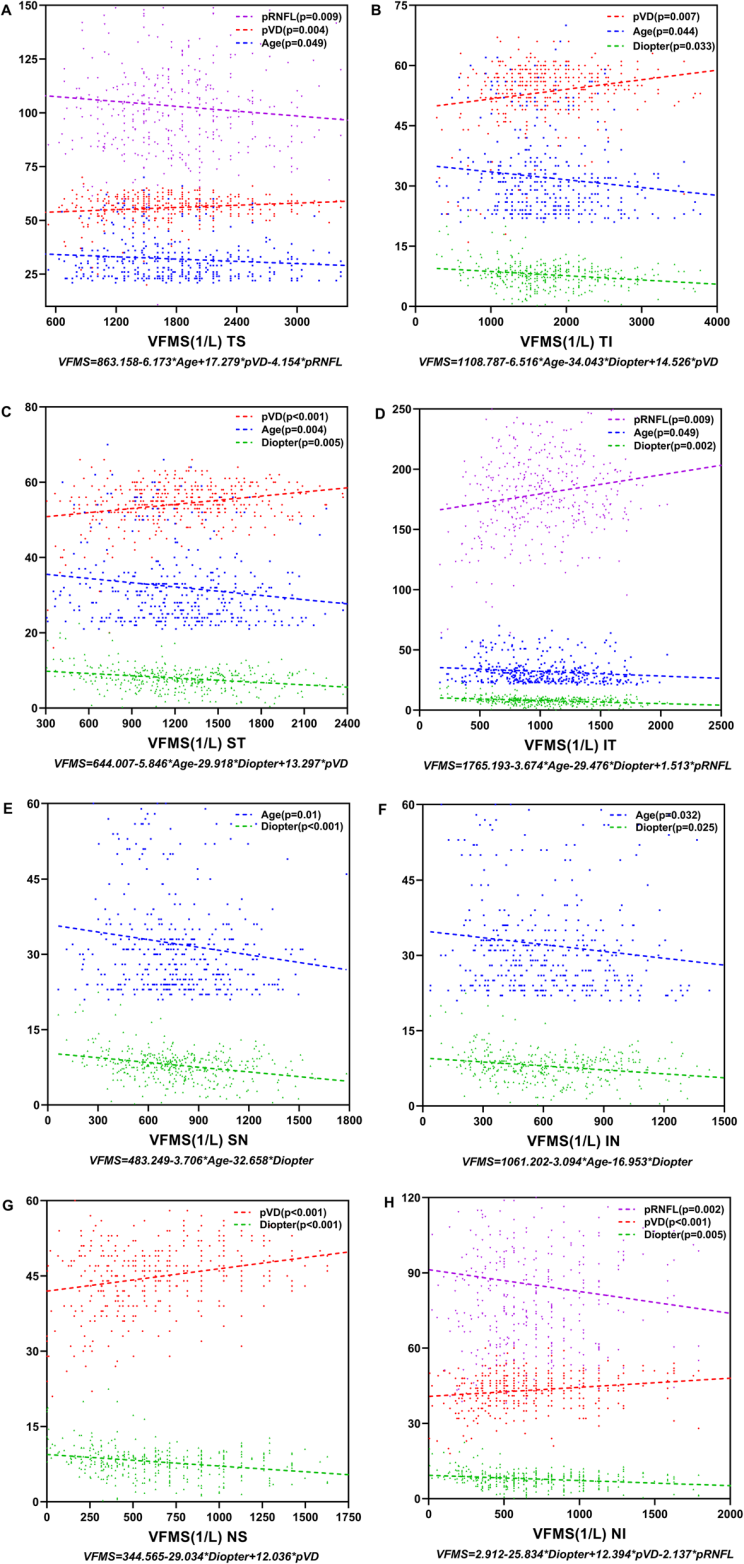
Multivariate regression analyses to identify factors associated with 8 peripapillary regional VFMS. Results of multivariate regression analyses showed a significantly positive relationships between peripapillary vessel density (pVD) and visual field mean sensitivity (VFMS) in myopic eyes, especially at the temporal and nasal quadrants (TS, TI, ST, NS and NI). Age and diopters were negatively correlated with VFMS. The relationships between pRNFL and the VFMS were not stable. In the TS and NI sectors, the pRNFL could negatively affected VFMS. While in the IT sector, the relationship between pRNFL and VFMS became positive. The linear regression equation for every regional VFMS was marked under the picture. **A** The VFMS in TS region was negatively correlated with age and pRNFL, while positively correlated with pVD. **B** In TI region, the VFMS was negatively correlated with age and refractive diopters, whereas positively correlated with pVD. **C** The VFMS in ST region was negatively correlated with age and refractive diopters, and positively correlated with pVD. **D** The VFMS in IT region was positively correlated with pRNFL, while negatively correlated with age and refractive diopters. **E** In SN region, the VFMS was negatively correlated with age and refractive diopters. **F** In IN region, the VFMS was negatively correlated with age and refractive diopters. **G **The VFMS in NS region was positively correlated with pVD and negatively correlated with refractive diopters. **H** The VFMS in NI region was positively correlated with pVD, while negatively correlated with refractive diopters and pRNFL. TS: temporal superior; TI: temporal inferior, ST: superior temporal, IT: inferior temporal, SN: superior nasal, IN: inferior nasal, NS: nasal superior, NI: nasal inferior

**Fig. 7:**
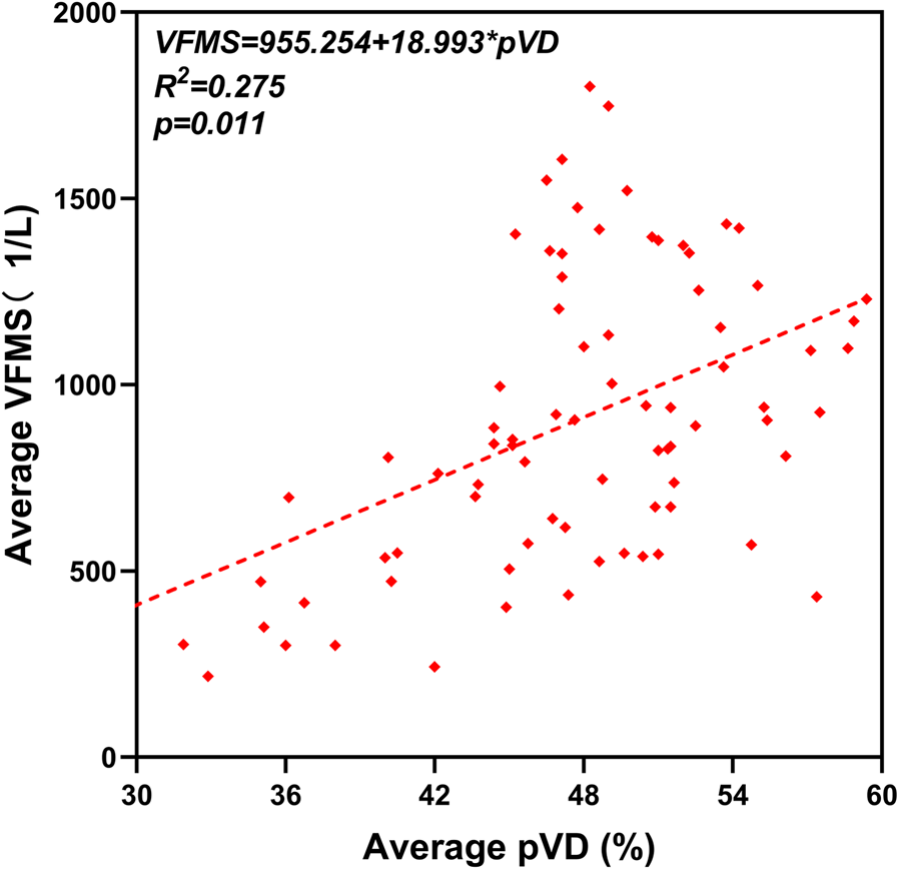
Multivariate regression analysis to identify factors associated with global VFMS in Group 4. Result of multivariate regression analysis showed a significant positive relationship between average peripapillary vessel density (pVD) and average visual field mean sensitivity (VFMS) in myopic eyes with SE ≤ −10D. The linear regression equation for average VFMS in Group 4 was marked with a correlation coefficient (R^2^) of 0.275

The results from the linear regression analyses to determine the factors associated with regional function according to the VFMS of myopic eyes are summarized in Tables 6 and 7. In Table 6, the univariate linear regression analysis was used to measure potential correlations between factors such as age, IOP, axial length, refractive error, average pRNFL and pVD with VFMS. The results showed that most of regional VFMS was correlated with age, axial length, refractive error and pVD (p < 0.05). While pRNFL was negatively correlated with VFMS in the TS and NI sectors. In Table 7, multivariate linear regression analysis was used with adjusting multiple factors for statistical analysis. The results of multivariate linear regression analysis demonstrated that age, refractive error diopters were negatively correlated with VFMS, whereas pVD has a positive association with VFMS (Fig. 6). We performed an extra linear regression analysis in group 4 to find out the factors associated with the average level of VFMS. Although in univariate linear regression analysis, the possible factors which associated with VFMS were axial length, refractive error and average pVD. In the following multivariate mode, only average pVD could positively affect visual function (Table 8, Fig. 7).

**Table 6:** Univariate regression analyses to identify factors associated with the visual field mean sensitivity (VFMS) in 8 zones

	Age			IOP			AL			Diopter			pVD			pRNFL		
	B	95% CI	P	B	95% CI	P	B	95% CI	P	B	95% CI	P	B	95% CI	P	B	95% CI	P
TS	−** 6.92**	**− 13.11 to − 0.74**	**0.028**	19.54	− 6.58 to 45.65	0.142	**− 62.72**	**− 106.20 to − 19.24**	**0.005**	**− 39.80**	**− 59.58 to − 20.02**	** < 0.001**	**16.80**	**7.06 to 26.54**	**0.001**	**− 2.84**	**− 5.57 to − 0.12**	**0.041**
TI	**− 7.93**	**− 14.24 to − 1.62**	**0.014**	7.92	− 18.80 to 34.64	0.56	**− 73.48**	**− 117.73 to − 29.22**	**0.001**	**− 45.20**	**− 65.30 to − 25.09**	** < 0.001**	**18.22**	**9.73 to 26.71**	** < 0.001**	0.20	− 2.68 to 3.08	0.891
ST	**− 6.65**	**− 10.78 to − 2.52**	**0.002**	9.15	− 8.42 to 26.73	0.307	**− 61.21**	**− 90.11 to − 32.32**	** < 0.001**	**− 37.50**	**− 50.54 to − 24.46**	** < 0.001**	**18.34**	**11.55 to 25.14**	** < 0.001**	1.29	− 0.06 to 2.65	0.061
IT	**− 5.74**	**− 9.55 to − 1.94**	**0.003**	− 12.28	− 28.42 to 3.85	0.135	**− 75.36**	**− 101.45 to − 49.27**	** < 0.001**	**− 40.50**	**− 52.30 to − 28.71**	** < 0.001**	**10.15**	**4.28 to 16.02**	**0.001**	**1.89**	**0.82 to 2.96**	**0.001**
SN	**− 4.43**	**− 7.34 to − 1.52**	**0.003**	3.71	− 8.67 to 16.08	0.556	**− 42.11**	**− 62.46 to − 21.76**	** < 0.001**	**− 28.90**	**− 38.00 to − 19.80**	** < 0.001**	**6.71**	**2.33 to 11.10**	**0.003**	0.29	− 0.58 to 1.17	0.511
IN	**− 3.90**	**− 6.78 to − 1.01**	**0.008**	− 9.10	− 21.28 to 3.15	0.145	**− 45.57**	**− 65.61 to − 25.53**	** < 0.001**	**− 23.64**	**− 32.77 to − 14.50**	** < 0.001**	**6.62**	**1.62 to 11.62**	**0.010**	0.46	− 0.46 to 1.38	0.325
NS	− 0.87	− 4.76 to 3.03	0.662	6.83	− 9.54 to 23.20	0.412	**− 44.25**	**− 71.38 to − 17.12**	**0.001**	**− 37.02**	**− 49.09 to − 24.95**	** < 0.001**	**16.44**	**10.63 to 22.25**	** < 0.001**	0.45	− 0.93 to 1.83	0.523
NI	**− 3.88**	**− 7.47 to − 0.29**	**0.034**	6.88	− 8.31 to 22.06	0.37	**− 40.53**	**− 65.71 to − 15.35**	**0.002**	**− 28.55**	**− 39.91 to − 17.19**	** < 0.001**	**10.24**	**5.05 to 15.42**	** < 0.001**	**− 1.28**	**− 2.49 to − 0.08**	**0.037**

The difference was statistically significant when p < 0.05 and significant values were in bold type

IOP, intraocular pressure; AL, axial length; pVD, peripapillary vessel density; pRNFL, peripapillary retinal nerve fibre layer

TS, temporal superior; TI, temporal inferior; ST, superior temporal; IT, inferior temporal; SN, superior nasal; IN, inferior nasal; NS, nasal superior; NI, nasal inferior

**Table 7:** Multivariate linear regression analyses to identify factors associated with the visual field mean sensitivity (VFMS) in 8 peripapillary zones

	Age			AL			Diopter			pVD			pRNFL			R^2^
	B	95% CI	P	B	95% CI	P	B	95% CI	P	B	95% CI	P	B	95% CI	P	
TS	**− 6.17**	**− 12.31 to − 0.04**	**0.049**	29.85	− 38.63 to 98.33	0.392	− 27.01	− 59.70 to 5.69	0.105	**17.28**	**5.59 to 28.97**	**0.004**	**− 4.16**	**− 7.29 to − 1.03**	**0.009**	0.072
TI	**− 6.37**	**− 12.55 t o − 0.19**	**0.044**	11.47	− 57.56 to 80.49	0.744	**− 35.67**	**− 68.44 to − 2.91**	**0.033**	**12.45**	**3.34 to 21.55**	**0.007**				0.076
ST	**− 5.85**	**− 9.81 to − 1.88**	**0.004**	11.03	− 33.22 to 55.28	0.624	**− 29.92**	**− 50.78 to − 9.06**	**0.005**	**13.30**	**6.16 to 20.43**	** < 0.001**				0.123
IT	**− 3.67**	**− 7.33 to − 0.01**	**0.049**	− 23.65	− 65.03 to 17.73	0.262	**− 29.48**	**− 48.48 to − 10.47**	**0.002**	− 0.70	− 7.28 to 5.89	0.836	**1.51**	**0.38 to 2.65**	**0.009**	0.136
SN	**− 3.71**	**− 6.51 to − 0.90**	**0.010**	19.59	− 11.72 to 50.90	0.219	**− 32.66**	**− 47.31 to − 18.01**	** < 0.001**	3.10	− 1.30 to 7.50	0.167				0.113
IN	**− 3.09**	**− 5.92 to − 0.27**	**0.032**	− 11.69	− 43.29 to 19.91	0.468	**− 16.95**	**− 31.80 to − 2.10**	**0.025**	2.16	− 3.07 to 7.38	0.417				0.077
NS				47.07	− 6.21 to 87.93	0.240	**− 29.03**	**− 41.53 to − 16.54**	** < 0.001**	**12.04**	**6.06 to 18.01**	** < 0.001**				0.120
NI	− 3.40	− 6.86 to 0.05	0.053	23.96	− 14.56 to 62.48	0.222	**− 25.83**	**− 44.00 to − 7.67**	**0.005**	**12.39**	**6.50 to 18.29**	** < 0.001**	**− 2.14**	**− 3.46 to − 0.82**	**0.002**	0.112

The difference was statistically significant when p < 0.05 and significant values were in bold type

AL, axial length; pVD, peripapillary vessel density; pRNFL, peripapillary retinal nerve fibre layer

TS, temporal superior; TI, temporal inferior; ST, superior temporal; IT, inferior temporal; SN, superior nasal; IN, inferior nasal; NS, nasal superior; NI, nasal inferior

R^2^, correlation coefficient

**Table 8:** Univariate and multivariate regression analyses to identify factors associated with the average visual field mean sensitivity (VFMS) in group 4

	Univariate			Multivariate			
	B	95% CI	P value	B	95% CI	P value	R^2^
Age	− 5.59	− 13.11 to 1.92	0.142				
AL (mm)	**− 88.48**	**− 149.50 to − 24.47**	**0.005**	− 21.124	− 92.37 to 50.12	0.557	
Diopter (D)	**− 64.64**	**− 93.07 to 36.21**	** < 0.001**	− 30.33	− 72.58 to 11.92	0.157	
Average pVD (%)	**28.01**	**16.36 to 39.66**	** < 0.001**	**18.99**	**4.55 to 33.44**	**0.011**	0.275
Average pRNFL (μm)	1.20	− 2.82 to 5.21	0.555				

The difference was statistically significant when p < 0.05 and significant values were in bold type

AL, axial length; pVD, peripapillary vessel density; pRNFL, peripapillary retinal nerve fibre layer

R^2^, correlation coefficient

## Discussion

**Fig. 8:**
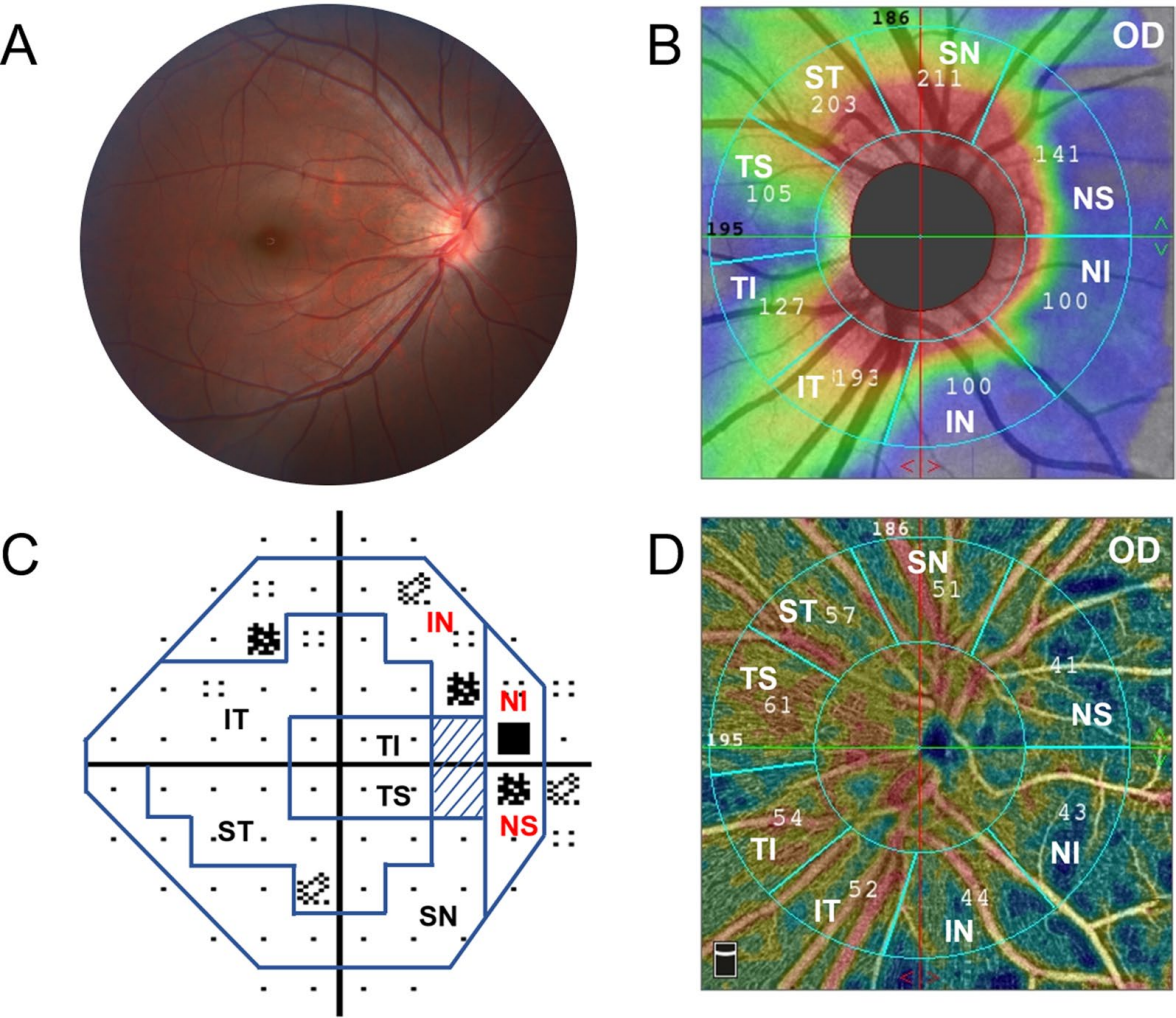
A representative case of structure–function relationship of high myopia. Peripapillary vessel density (pVD) assessed by optical coherence tomography angiography (OCT-A) shows a better correlation with the visual field (VF) damage than the peripapillary retinal nerve fibre layer (pRNFL) assessed by spectral domain optical coherence tomography (SD-OCT). A 25-year-old woman presented with high myopia in the right eye whose spherical equivalent (SE) was −8.25DS and axial length was 26.15 mm. **A** The fundus photography showed no obvious abnormalities in the patient's optic disc structure and no glaucomatous optic disc changes (such as localized or diffuse rim thinning, disc hemorrhage, notch in the rim, and vertical cup-to-disc ratio higher than that of the other eye by more than 0.2). **B** The nerve fibre layer image performed with SD-OCT showed no decrease in the 8 peripapillary regions. **C** The VF damage happened in the nasal inferior (NI), nasal superior (NS) and inferior nasal (IN) regions which were highlighted with red color. **D** The pVD showed focal reduction also in the NI,NS and IN regions corresponding VF defect

With the advances in imaging techniques, peripapillary assessment remains a challenge in highly myopic eyes because of structural variability. Not only structure alteration, but high myopia can also result in functional impairment, sometimes similar to glaucomatous VF damage [5]. Therefore, it’s important to investigate the relationships between structure and function in high myopia. This prospective study, which enrolled two hundred and twenty-two healthy adults in 18 months, was the first cross-sectional study evaluating the globe and regional structure–function relationships in healthy myopic eyes. According to this study, the significant decrease of VFMS was observed only in high myopia with SE ≤ − 10.00D. The average pVD assessed by OCTA showed a significant association with VFMS in eyes with SE ≤ − 8.00D while the pRNFL was not statistically correlated with VFMS in our study. Figure 8 shows a representative case in which the pVD reflects VF damage better than pRNFL in highly myopic eyes. Our findings may suggest that pVD rather than pRNFL could be a useful and sensitive test for monitoring functional changes in myopic eyes. It is interesting to note that, a reduced pVD due to myopic change was not observed with SE > − 8.00D. In those eyes with SE between − 8.00D to − 10.00D, only a significant decrease in pVD was observed while the VFMS was not statistically changed. The pVD reduction accompanied by VFMS decrease will appear when SE ≤ − 10.00D. We speculate that the microvascular change happened earlier than the functional change in highly myopic eyes. These results highlight that pVD assessed by OCTA is expected to be a sensitive indicator for monitoring visual function in high myopia.

The elongation of axial length and deformation of the eyeball in high myopia will not only result in a range of retinal and choroidal lesions [30, 31] but also make the assessment of the peripapillary structure difficult. Several studies have reported that the pRNFL is significantly thinner in highly myopic eyes [29–31]. However, both Kim et al. [32] and Kang’s [33] research have demonstrated that highly myopic eyes have a significantly thicker temporal RNFL and thinner RNFL in the non-temporal sectors compared with eyes with low myopia. In the present study, after ocular magnification adjustment, the average pRNFL thickness had no statistically difference despite the refractive error of myopia. Whereas in highly myopic eyes there had a significantly thicker temporal superior and temporal inferior pRNFL and significantly thinner nasal superior pRNFL than those mild-to-moderate myopic eyes (Table 2). This kind of pRNFL distribution pattern is contributed with the temporally tilted optic disc which makes the nasal half of the optic disc elevates anteriorly, and the temporal half of the optic disc depresses posteriorly [34]. Therefore, this kind of pRNFL structure alteration makes it difficult to monitor changes in visual function with the pRNFL assessment in highly myopic eyes. In our study, there had no significant correlation between average pRNFL and VFMS among all the four groups in our study.

In our study, the average pVD were significantly reduced with the increase of negative myopic diopter (Table 3). In regional analysis, this trend was obvious especially in those subjects whose refractive error ≤ − 8.00D (Fig. 4). At the same time, the pVD in most of the regions showed no difference between group 1 and group 2 except for the NS and NI sectors. The result documented that regional pVD showed a significant downward trend in those with SE ≤ − 8.00D and was relatively stable in the eyes with SE > − 8.00D. Several studies used OCTA to investigate the changes of retinal microvascular in myopia subjects [3, 6]. He et al. have reported vessel density was significantly lower in eyes with high myopia than in eyes with emmetropia and mild to moderate myopia [35]. Wang et al. also reported that eyes with high myopia have a decreased pVD compared with emmetropic eyes [36]. Although the results of these studies suggested a decrease of pVD in highly myopic populations whose SE ≤ − 6.00D, but in our study the regional pVD wouldn't demonstrate a significant reduce until SE ≤ − 8.00D. The reduction was observed in five out of eight sectors in group 3 except for the TS, TI and SN sectors. Meanwhile all the regional pVD was decreased significantly in group 4. These results suggested that the regional peripapillary vascular structure altered significantly in eyes with SE ≤ − 8.00D (Table 3, Fig. 4). These findings implied that pVD may be more sensitive to myopic changes than pRNFL and the differences in the pVD and pRNFL profiles might result in significantly different in the pVD-VFMS association and the pRNFL–VFMS association.

Previous studies have reported that visual field defects developed and progressed in highly myopic eyes [4, 37]. But there haven’t consistent findings on the pattern of visual field damage due to high myopia, not to mention the relationships between structure and function. In this study, the VFMS in group 4 (SE ≤ − 10.00D) were statistically lower than other three groups not only in the average level but also in the eight functional regions (Table 4). The differences of regional VFMS between group 1,2 and 3 were not as obvious as that in pVD (Fig. 4). Only in functional zone 4 which corresponding to structure region IT, statistical difference was observed between group 1 and group 3. At the same time the significant differences of pVD were found in most of the regions between group 1 and group 3. In group 4, the decreasing trends of pVD are in line with the decreasing trends of VFMS (Fig. 3). Presumably, the peripapillary vessel density may change earlier than the function does in highly myopic eyes. Therefore, in the following correlation analysis, positive associations were showed between average pVD and VFMS in eyes with refractive error ≤ − 8.00D (r = 0.184 & p = 0.042 in group 3, r = 0.476 & p < 0.001 in group 4, Table 5). Furthermore, the pVD-VFMS relationship in group 4 was statistically stronger compared with the relationship in group 3 (p < 0.001, Steiger’s z test). In regional analysis, there were three zones (2, 7 and 8) in group 3 and all the eight zones in group 4 where positive relationships were observed between pVD and VFMS (Fig. 5). We also observed an interesting phenomenon, weak to moderate positive relationships between pVD and VFMS were showed at the horizontal quadrants (TI, NS and NI) in group 1, 2 and 4. At the vertical quadrants (ST, IT, SN and IN), it was not until the high myopia group with SE ≤ − 10.00D that a significant correlation between pVD and VFMS were observed. The results of linear regression analysis also showed a positive relationship between pVD and VFMS in the temporal and nasal quadrants in myopic eyes (Fig. 6).

Myopia is a known risk factor for glaucoma, it can lead to structural and functional defects that cannot be easily distinguished from those caused by glaucoma [38]. Many researchers have been explored to try to find out how to identify the differences of VF damage caused by glaucoma or high myopia. Investigating the relationships between structure and function is one of the main research directions. A reduced pVD due to myopic change may serve as a confounding factor in evaluating the vasculature-function relationship in glaucoma. Although there is a moderate relationship between structure and function in glaucoma in emmetropic patients, the relationship between RNFL and VF was relatively weak in glaucomatous eyes with high myopia [39, 40]. Shin et al. [6] have reported a stronger relationship between microvascular structure and VF in highly myopic glaucoma patients and they found the vascular parameters seemed to be less affected by myopic changes. Whereas in our study, along with the increase of negative refractive error and axial length, the globe and regional pVD decreased significantly especially with those SE ≤ − 8D. Although the microvasculature-function relationship was not obvious in eyes with -6D≧SE > − 8D group, the pVD-VFMS relationship was significant in eyes with SE ≤ − 10D group. This might be explained by the different vascular pattern of high myopia and glaucoma damage. According to previous research, the microvascular network may be stretched in highly myopic eyes, while the pattern of pVD loss caused by glaucoma may appear as a dropout [3, 41]. Therefore, myopic stretching has a lesser decrease of pVD compared with glaucomatous microvascular dropout. So, in highly myopic glaucoma patients, the dropout of microvasculature caused by glaucoma might cover the decrease of pVD caused by high myopia. Another possible reason for the different results of our study from others may due to the different grouping method. We grouped high myopia into three subgroups according to the diopter, whereas most previous studies wouldn’t classified subgroups among high myopia. Therefore, we observed a rather different pVD distribution pattern and a strong pVD-VFMS relationship in those eyes with SE ≤ − 10D (Fig. 7).

We acknowledge this study has several limitations that should be taken into account when the results are interpreted. This cross-sectional design does not allow a causality examination between vascular structure and visual function and the sample size might have led to misleading results in gender-specific analysis. Although we have observed some regional differences of peripapillary alterations due to myopia in pVD and VFMS, further longitudinal studies are required to reveal this regional pattern.

In conclusion, we found and characterized peripapillary vascular density alterations in highly myopic eyes, which appeared to occur in a diffuse manner around the optic disc in eyes with SE ≤ − 8.00D. A significant decrease of VFMS was observed in high myopia with SE ≤ − 10.00D. The average pVD assessed by OCTA showed a significant association with VFMS in eyes with SE ≤ − 8.00D, meanwhile the pRNFL was not statistically correlated with VFMS. Therefore, the pVD measurement by OCTA could be a sensitive and useful method for monitoring myopic functional change rather than pRNFL especially in the temporal and nasal quadrants.

## Data Availability

All data generated or analyzed during this study are included in this published article.
